# Beyond Redox Synergy: Role of Trivalent Cations in Ni-Based Layered Double Hydroxides for Peroxydisulfate Activation

**DOI:** 10.1021/acsomega.6c07992

**Published:** 2026-10-21

**Authors:** Edgar Oswaldo Leyva Cruz, Refugio Rodríguez-Vázquez, Deyanira Angeles-Beltrán

**Affiliations:** † 681581 Centro de Investigación y Estudios Avanzados del Instituto Politécnico Nacional, Doctorado en Ciencias (Nanociencias y Nanotecnología), Departamento de Investigación y Estudios Multidisciplinarios, Av. Instituto Politécnico Nacional 2508, Col. San Pedro Zacatenco, Delegación Gustavo A. Madero, Ciudad de México C.P. 07360, México; ‡ Centro de Investigación y de Estudios Avanzados del Instituto Politécnico Nacional, Departamento de biotecnología y Bioingeniería, Av. Instituto Politécnico Nacional 2508, Col. San Pedro Zacatenco, Delegación Gustavo A. Madero, Ciudad de México C.P. 07360, México; § Área académica de Química, Departamento de Ciencias Básicas, Universidad Autónoma Metropolitana Unidad Azcapotzalco, Av. San Pablo No. 420 Col. Nueva del Rosario, Azcapotzalco, Ciudad de México C.P. 02128, México

## Abstract

The persistence of fluoroquinolone antibiotics in aquatic environments represents a growing environmental concern and requires efficient and scalable treatment strategies. In this study, ciprofloxacin degradation was investigated through persulfate activation by using Ni-based layered double hydroxides (LDHs). NiFe-LDHs with different molar ratios (1:1, 2:1, and 3:1) were synthesized and compared with NiAl-LDH to elucidate the role of the trivalent metal in the catalytic performance. A 2^2^ factorial design was employed to evaluate the influence of the catalyst dosage and persulfate volume. All catalysts exhibited rapid removal, reaching approximately 57% removal within 20 min and up to 87% removal after 120 min. Increasing the Ni content did not improve the overall removal performance, indicating that activity is not directly governed by the amount of Ni present. Kinetic analysis followed pseudo-second-order behavior, while factorial results indicated that catalyst dosage had a stronger influence than the persulfate concentration. Mass spectrometry confirmed the disappearance of the parent ion (*m*/*z* 332), indicating an effective transformation of ciprofloxacin. Radical scavenger experiments indicated a greater contribution of radical species in the Fe-containing materials, whereas NiAl-LDH showed a more adsorption-driven removal pathway. No synergistic enhancement between Ni and Fe was observed, as NiAl-LDH exhibited comparable or superior performance despite containing the non-redox-active Al^3+^ center. Density functional theory (DFT) calculations revealed that Ni centers play a dominant role in persulfate activation, whereas the trivalent metal primarily modulates the electronic structure and the reaction pathway of the catalyst. These findings challenge the conventional assumption that the presence of multiple redox-active metal centers necessarily enhances the catalytic performance in LDH systems and highlight the critical role of the local electronic environment in designing efficient catalysts for environmental applications.

## Graphical abstract

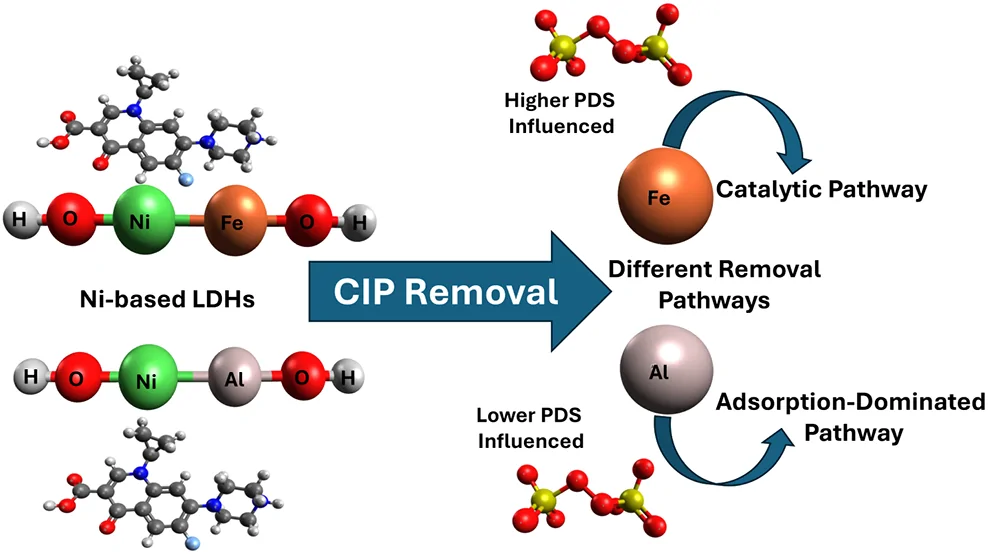

## 1 Introduction

Water contamination by pharmaceuticals, commonly referred to as emerging contaminants, has become a growing concern due to their potential impact on diverse ecosystems.^1^ These compounds can disrupt beneficial bacterial communities and, through continuous exposure, promote the development of bacterial resistance to antibiotics.^2^

A major challenge associated with pharmaceuticals is their large-scale production. Globally, pharmaceutical manufacturing generates wastewater with high concentrations of organic species (500–6500 m^3^), often containing biotoxic substances, fermentation byproducts, organic solvents, and metabolites.^3^

Among these pollutants, ciprofloxacin (Figure 1) has gained significant attention, as it is reported to be the second-most widely used antibiotic. Two critical aspects are alarming: the high amounts found in wastewater sources and the fact that over 75% of the drug remains unmetabolized and is excreted.^4^ Consequently, ciprofloxacin residues contribute to the pollution of surface water, groundwater, and drinking water supplies. This persistence highlights the urgent need for effective removal methods to prevent antibiotics from reaching potable water sources.

**Figure 1:**
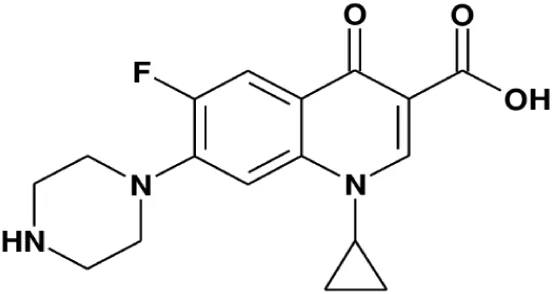
Ciprofloxacin chemical structure.

Conventional wastewater treatment processes are generally ineffective in removing these contaminants,^5^ which underscores the importance of advanced oxidation processes (AOPs). AOPs rely on the in situ generation of highly reactive oxidizing species such as hydrogen peroxide (H_2_O_2_), ozone (O_3_), and persulfate (SO_4_^2–^/S_2_O_8_^2–^), offering environmentally friendly, efficient, and cost-effective solutions.^6^

Among AOPs, persulfate activation has recently attracted attention due to its Fenton-like reaction mechanism. In this process, persulfate (S_2_O_8_^2–^) is activated to produce sulfate radicals (SO_4_•−), thereby enabling rapid degradation of organic pollutants. Transition-metal-based catalysts are typically required, since their redox cycling facilitates radical generation with different efficiencies (Ni^2+^ < Fe^3+^ < Mn^2+^ < V^3+^ < Ce^3+^ < Fe^2+^ < Ru^3+^ < Co^2+^).^7^ These metal ions alternate between oxidation states, producing radicals, as illustrated in reactions R1–R3:^8^

R1 $$S_{2}{O_{8}}^{2-\;}{+\;M}^{2+}\to M^{3+}+{{\mathrm{SO}}_{4}}^{2-}+{\mathrm{SO}}_{4}\mathrm{•‐}$$

R2 $$M^{3+}+\mathrm{Red}\to M^{2+}+\mathrm{Ox}$$

R3 $${\mathrm{SO}}_{4}\mathrm{•‐}+\mathrm{Organic compound}\to \mathrm{Oxidation products}$$

where Red and Ox denote the reduced and oxidized forms of a generic electron donor, respectively.

Different activation methods for persulfate have been reported, including thermal,^9^ electrochemical,^10^ photoassisted,^11^ and catalytic activation.^12^ Among these, catalytic activation has drawn particular attention due to its feasibility and effectiveness as a suitable process in which different catalyst materials are employed.

A relevant aspect of persulfate activation is the use of catalysts containing two or more redox-active metals. Several studies have reported that multimetallic systems often exhibit improved performance in comparison with single-metal catalysts, primarily due to synergistic redox interactions that accelerate radical generation.^13^ Iron is widely recognized as a fundamental component of advanced oxidation processes, and its catalytic activity can be further enhanced by incorporating a secondary transition metal, such as nickel.^14^

Layered double hydroxides (LDHs) are promising catalysts, owing to their versatility and tunable composition. They have been studied in diverse applications, such as oxygen reduction with CoFe,^15^ water splitting with NiCo,^16^ quinoline synthesis with CuMgMn,^17^ polyester synthesis with MgAlTi,^18^ and degradation of sulfamethoxazole with CoMn.^19^ LDHs (Figure 2) possess a crystalline lamellar structure, composed of divalent and trivalent cations and hydroxyl groups, with interlayer anions, such as CO_3_^2–^, NO_3_^–^, or SO_4_^2–^ balancing the positive charge.^20^ Particularly for environmental catalysis, this highly ordered two-dimensional structure provides an exceptionally high specific surface area, abundant and uniformly distributed active sites across the brucite-like layers, and structural stability that favors interaction with oxidizing agents.

**Figure 2:**
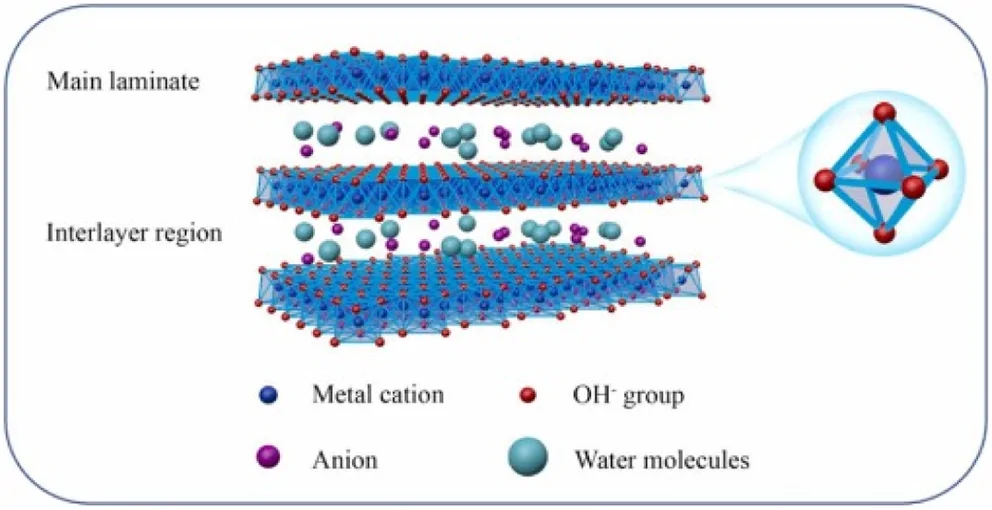
General structure of Layered Double Hydroxide.^21^

In this work, ciprofloxacin removal was investigated through persulfate activation catalyzed by NiFe-LDHs. The materials were synthesized via coprecipitation with varying molar ratios (1:1, 2:1, and 3:1) and characterized by X-ray diffraction, SEM-EDS, and N_2_ physisorption. Ultimately, this study aims to evaluate the potential synergistic effect between Ni and Fe during persulfate activation, elucidating the specific role of Fe by comparing the catalytic performance against a non-redox-active NiAl-LDH. DFT calculations were additionally employed to rationalize the observed catalytic behavior from an electronic-structure perspective.

## 2 Methodology

### 2.1 Chemicals

Nickel­(II) nitrate (Ni­(NO_3_)_2_·6H_2_O, Mayer, 98%), iron­(III) nitrate (Fe­(NO_3_)_3_·9H_2_O, Meyer, 98%), aluminum­(III) nitrate (Al­(NO_3_)_3_·9H_2_O, Meyer, 98%), potassium persulfate (K_2_S_2_O_8_, Aldrich, 99%), sodium carbonate (Na_2_CO_3_, Mallinckrodt, 100%), sodium hydroxide (NaOH, Meyer, 97%), *tert*-butanol, and methanol were purchased from Sigma-Aldrich.

### 2.2 Synthesis of Ni/Fe Layered Double Hydroxide (LDH)

The appropriate amounts of nickel and iron salts were used to obtain Ni:Fe molar ratios of 1:1, 2:1, 3:1, and 3:1 for (Ni:Al). Nickel nitrate Ni­(NO_3_)_2_·6H_2_O and iron nitrate Fe­(NO_3_)_3_·9H_2_O and Al­(NO_3_)_3_·9H_2_O for the NiAl LDH were dissolved in 100 mL of deionized water. The resulting solution was added dropwise to 100 mL of a basic solution containing NaOH and Na_2_CO_3_, with constant stirring at room temperature, thereby promoting the coprecipitation of the solid phase. During this process, a 3 M NaOH solution was added to maintain pH = 10. Subsequently, the mixture was thermally treated in an oven at 80 °C for 24 h. Finally, the solid was recovered by filtration, washed several times with deionized water, and dried at 90 °C overnight.

### 2.3 Material Characterization

The materials synthesized were characterized by XRD (Philips X’Pert diffractometer with Cu Kα radiation), SEM-EDS (SUPRA 55 VP Zeiss Scanning Electron Microscope), BET (ASAP 2020 Micromeritics surface area and porosity analyzer), and Raman spectroscopy (Raman inVia Renishaw).

### 2.4 Ciprofloxacin Removal

The ciprofloxacin removal experiments were conducted using a 30 mg/L ciprofloxacin solution; then, 25 mL of this solution was mixed with 0.5 mL of a 0.1 M potassium persulfate solution and 20 mg of catalyst.

During removal, the mixture was stirred continuously at room temperature. The reaction progress was monitored every 20 min by withdrawing aliquots, which were centrifuged for 1 min before analysis using a UV–vis spectrophotometer (Shimadzu UV-1800).

The removal of ciprofloxacin was monitored by UV–vis spectroscopy at the maximum absorbance (λmax = 270 nm). All experiments were performed in triplicate to ensure reproducibility.

### 2.5 DFT Theoretical Study

Density functional theory (DFT) calculations were performed to investigate the electronic properties of Ni-based layered double hydroxide (LDH) catalysts (NiFe-LDH and NiAl-LDH) and their thermodynamic potential to activate persulfate. All quantum chemical simulations were carried out using the ORCA software package (version 6.1.1).^22^ Representative tetranuclear molecular clusters were employed as simplified structural models to capture the local coordination environments of the metal-hydroxide active sites.

Geometry optimizations and electronic-structure calculations were conducted using the B3LYP hybrid functional in combination with the def2-SVP basis set. Noncovalent, long-range dispersion interactions were explicitly accounted for using Grimme’s D3 correction with Becke–Johnson damping (D3BJ).^23^ Given that the intended environmental application takes place in an aqueous matrix, bulk solvent effects were implicitly modeled using the Conductor-like Polarizable Continuum Model (CPCM) parametrized for water, which is critical for an accurate description of the electronic density in transition-metal complexes.

Owing to the presence of open-shell transition-metal centers Ni^2+^ and Fe^3+^ in the structural lattice, the local spin state plays a crucial role in dictating the ground-state electronic structure. Consequently, a systematic multiplicity scan was performed for each of the isolated cluster models. Spin multiplicities ranging from low-spin to high-spin configurations were evaluated to identify the electronic state with the lowest total self-consistent-field (SCF) energy.

From the identified ground-spin states, the frontier molecular orbitals (HOMO and LUMO), HOMO–LUMO energy gaps (*ΔE_gap_*) (eq 4), Hirshfeld atomic charges, and adsorption energies (*ΔG_ads_*) were extracted (eq 5). Subsequently, the cooperative interaction between the optimized LDH active sites and the persulfate anion (S_2_O_8_^2–^) was evaluated through unconstrained geometry optimizations of the corresponding oxidant–cluster complexes. All initial molecular architectures, optimized structures, and molecular orbital boundaries were visualized and prepared using Avogadro (version 1.2.0).

1 $$\Delta E_{gap}=E_{\mathrm{LUMO}\;}-E_{HOMO}$$

2 $$\Delta G_{ads}=G_{S_{2}{O_{8}}^{-2}-Cluster\;}-\left(G_{Cluster}+G_{S_{2}{O_{8}}^{-2}}\right)$$

### 2.6 Experimental Design

A 2^2^ factorial experimental design was conducted to evaluate the effects of catalyst mass and potassium persulfate solution volume on ciprofloxacin removal after 20 min of reaction (Table 1). This design allows for the assessment of both main and interaction effects between the factors. All experiments were performed in triplicate to ensure reproducibility.

**Table 1:** Factors and Their Corresponding Levels Applied in the 2^2^ Factorial Experimental Design for Ciprofloxacin Removal

Factors	Levels	
	–1	+1
Catalyst dosage (mg)	20	40
Persulfate volume solution (mL)	0.5	1

## 3 Results and Discussion

### 3.1 Material Characterization

#### 3.1.1 X-ray Diffraction

The materials were characterized by X-ray diffraction (XRD), revealing the characteristic reflections of the two-dimensional hydrotalcite-like structure. For the NiFe samples, diffraction peaks were observed at 2θ values of 11.8°, 23.7°, 34.7°, 39.4°, 46.8°, 60.3°, 61.5°, and 65.5° (Figure 3), which are consistent with the typical LDH phase reported in the literature.

**Figure 3:**
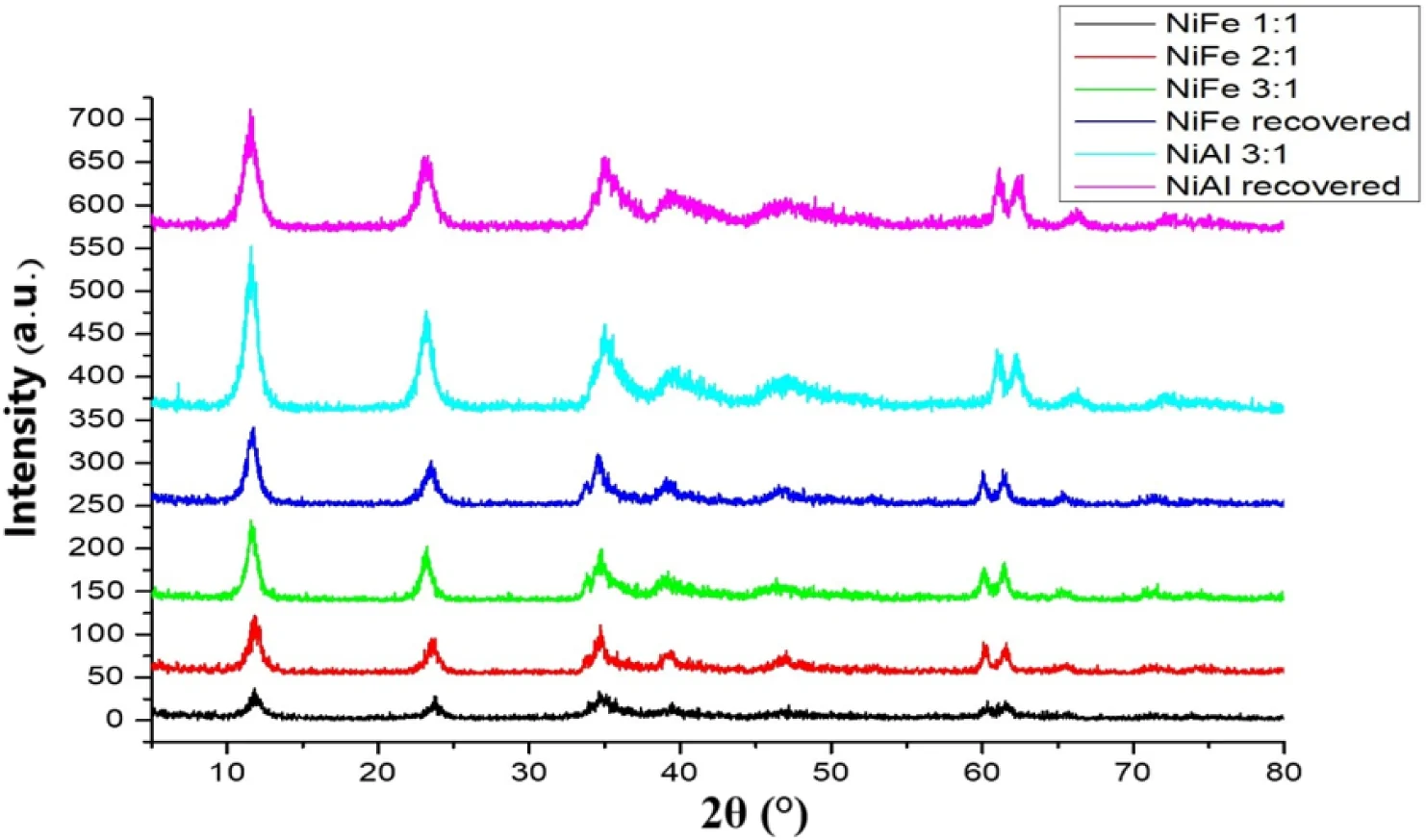
X-ray diffraction (XRD) patterns of the synthesized NiFe and NiAl layered double hydroxides before and after catalytic persulfate activation.

The average crystallite sizes, estimated using the Scherrer equation (Table 2), confirmed the nanocrystalline nature of all materials, with the NiFe sample prepared at a 2:1 molar ratio exhibiting the smallest crystallite size. The NiAl sample showed slightly higher crystallinity; however, its diffraction peaks and positions were comparable to those of the NiFe-based materials, indicating the successful formation of the LDH structure. Notably, both NiFe and NiAl samples synthesized with a 3:1 molar ratio exhibited very similar crystallite sizes.

**Table 2:** Average Crystallite Size for the Catalyst Studied

Elements ratio	Average crystallite size (nm)
NiFe 1:1	29.91
NiFe 2:1	19.85
NiFe 3:1	32.31
NiAl 3:1	37.24

Figure 3 presents the XRD patterns of the recovered NiFe(3:1) and NiAl(3:1) catalysts after the persulfate activation reaction. The diffraction profiles remain essentially unchanged compared to those of the fresh materials, showing the same characteristic reflections with only minor variations in the peak intensity. These results confirm that the hydrotalcite-like structure is preserved after use, indicating good structural stability under reaction conditions.

As summarized in Table 3, the comparison of the characteristic diffraction peak positions (2θ) and corresponding interplanar distances (*d*-spacing) for the four synthesized materials shows a consistent set of reflections characteristic of the layered double hydroxide (LDH) structure. The basal and nonbasal reflections were observed across all compositions, with only minor variations in their 2θ positions and *d*-spacing values.^21^ In particular, NiFe-LDH 1:1 exhibits the same characteristic diffraction reflections observed for NiFe-LDH 2:1, NiFe-LDH 3:1, and NiAl-LDH, supporting the assignment of the four materials to the LDH structural family. This observation is particularly relevant for the NiFe-LDH 1:1 composition, whose relatively high Fe^3+^/(Ni^2+^ + Fe^3+^) ratio could otherwise raise questions regarding the preservation of the LDH structure. The absence of additional diffraction reflections attributable to crystalline secondary phases further supports the structural assignment based on XRD. Nevertheless, small variations in peak positions and *d*-spacing values are observed among the compositions, reflecting changes in the local structural parameters associated with the incorporation of Fe^3+^ or Al^3+^ into the hydroxide layers.

**Table 3:** Experimental XRD Peak Positions 2θ and Interplanar *d*-Spacings for the Synthesized NiAl-LDH and NiFe-LDH Materials

	NiAl (3:1)		NiFe (3:1)		NiFe (2:1)		NiFe (1:1)	
No. Peak	Pos. 2θ (°)	*d*-spacing (Å)	Pos. 2θ (°)	*d*-spacing (Å)	Pos. 2θ (°)	*d*-spacing (Å)	Pos. 2θ (°)	*d*-spacing (Å)
1	11.60	7.63	11.65	7.60	11.88	7.45	11.86	7.46
2	23.22	3.83	23.20	3.83	23.63	3.77	23.73	3.75
3	35.10	2.56	34.66	2.59	34.73	2.58	34.83	2.58
4	39.79	2.27	38.98	2.31	39.25	2.30	39.42	2.29
5	47.25	1.92	46.43	1.96	46.85	1.94	46.82	1.94
6	61.03	1.52	60.12	1.54	60.21	1.54	60.30	1.53
7	62.24	1.49	61.43	1.51	61.52	1.51	61.63	1.50
8	66.04	1.41	65.34	1.43	65.43	1.43	65.51	1.42

#### 3.1.2 Raman Spectroscopy

The Raman spectra of Fe-based materials display multiple bands in the 200–500 cm^–1^ region, typically attributed to M–O and mixed M­(II)–O–M­(III) vibrational modes characteristic of layered double hydroxide structures. As the Ni content increases, these bands become more intense and better defined (Figure 4), suggesting modifications in the local metal–oxygen coordination environment consistent with changes in the Ni:Fe ratio.

**Figure 4:**
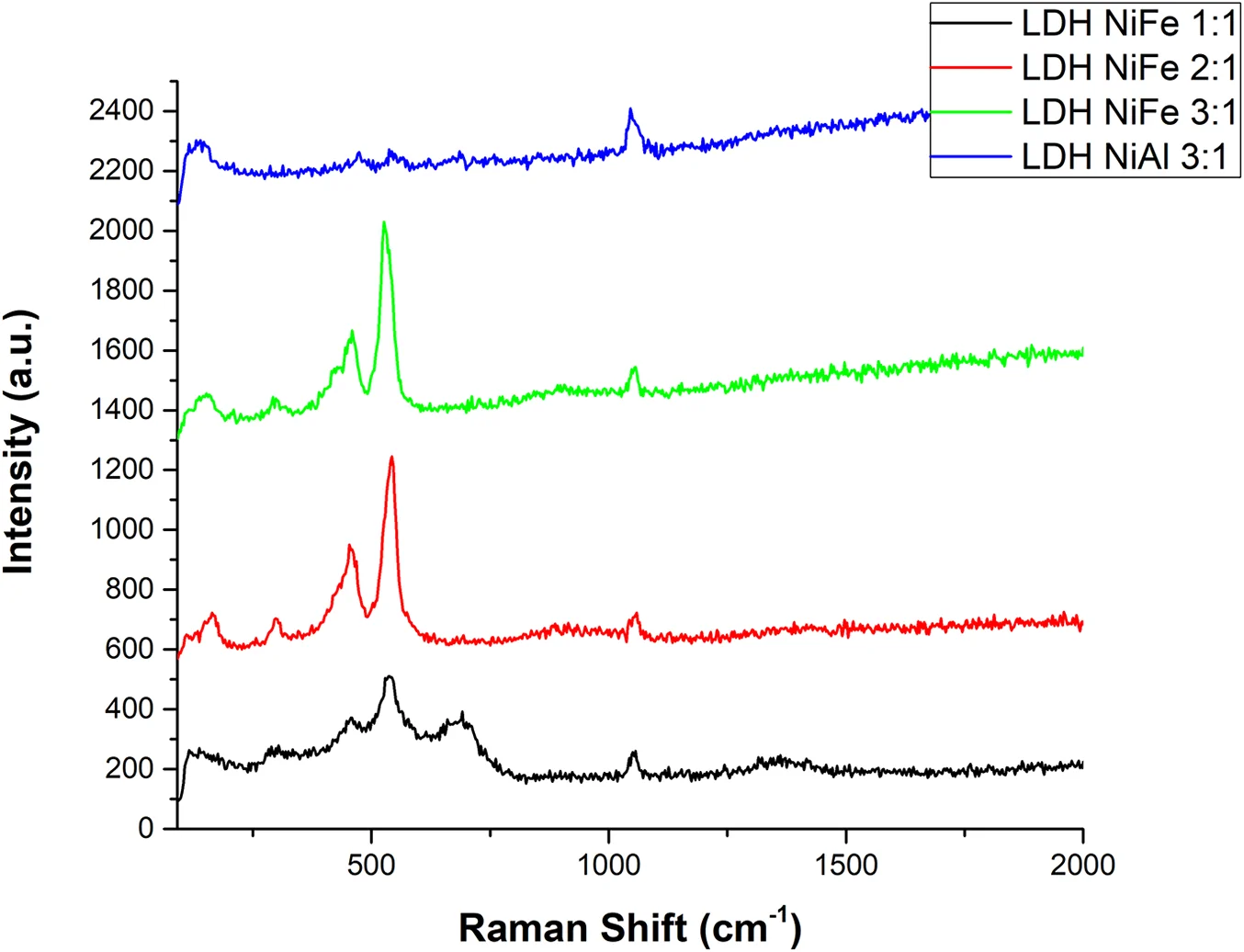
Raman spectra of fresh NiFe and NiAl LDHs, highlighting the structural variations with different metal molar ratios.

In contrast, the Al-based catalyst exhibits only one weak band in this region, which may reflect differences in lattice dynamics associated with the redox-inactive nature of aluminum compared to that of iron.

It is worth noting that NiFe 1:1 and NiAl 3:1 exhibit comparatively less defined spectral features in this region. While band definition and intensity can be influenced by multiple factors—including crystallinity, particle size, local coordination environment, phase composition, and signal normalization conditions—broader or weaker bands have in some cases been tentatively associated with changes in structural organization in related oxide systems.^24^ Although no direct quantification of defect density was performed in this study, these spectral observations are noted as potentially consistent with differences in local structural order, which may warrant further investigation.

#### 3.1.3 SEM-EDS

The NiFe and NiAl materials exhibit a laminar morphology typical of that of double-layered hydroxides. This is consistent with XRD analysis, which does not exhibit a significant difference between Fe- and Al-containing materials (Figure 5a–d).

**Figure 5:**
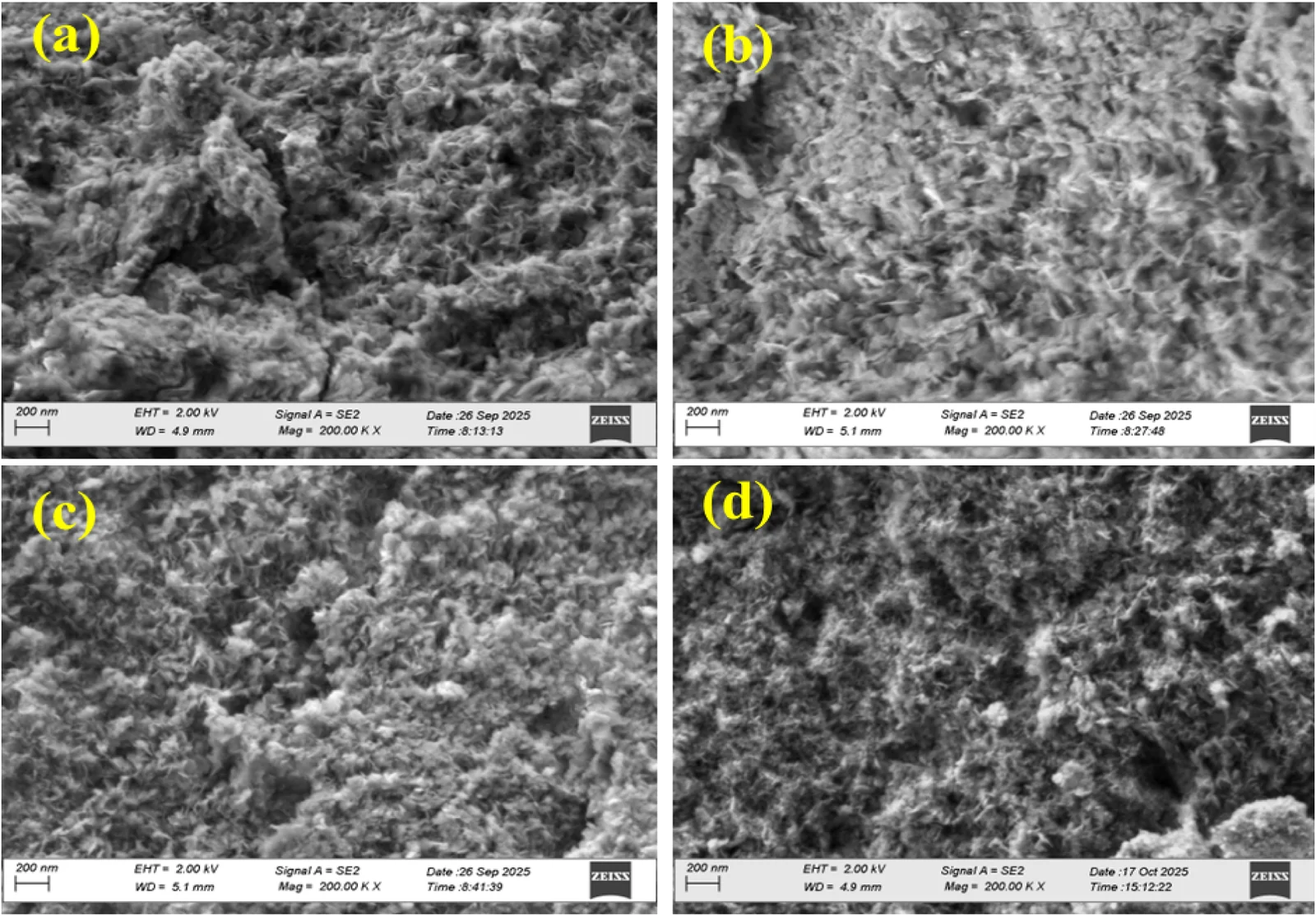
Micrographs of Ni-based materials: (a) NiFe 1:1, (b) NiFe 2:1, (c) NiFe 3:1, and (d) NiAl 3:1.

The elemental mapping analysis (Figure 6) and EDS analysis (Table 4) reveal distinct elemental distribution behaviors among the synthesized materials. The NiFe sample prepared at a 1:1 nominal Ni:Fe molar ratio exhibited the lowest Ni content (11.99 wt %) and the highest Fe content (28.41 wt %) among the NiFe materials. Consistently, the Ni elemental map showed a less homogeneous distribution with several regions of lower Ni signal, whereas Fe exhibited a comparatively uniform distribution across the analyzed surface. Increasing the nominal Ni:Fe ratio to 2:1 and 3:1 resulted in progressively higher Ni contents (32.67 and 52.66 wt %, respectively) and lower Fe contents (19.34 and 16.93 wt %, respectively). The corresponding elemental maps showed a more homogeneous Ni distribution at higher Ni:Fe ratios, although some regions of lower intensity remained visible in the 2:1 sample. This trend is consistent with the XRD and Raman spectroscopy results, as the 1:1 material exhibited the lowest crystallinity and a reduced Raman intensity. Overall, the EDS and elemental mapping results indicate that increasing the nominal Ni:Fe ratio was accompanied by an increase in the detected Ni content and a more homogeneous Ni distribution across the analyzed surface.

**Figure 6:**
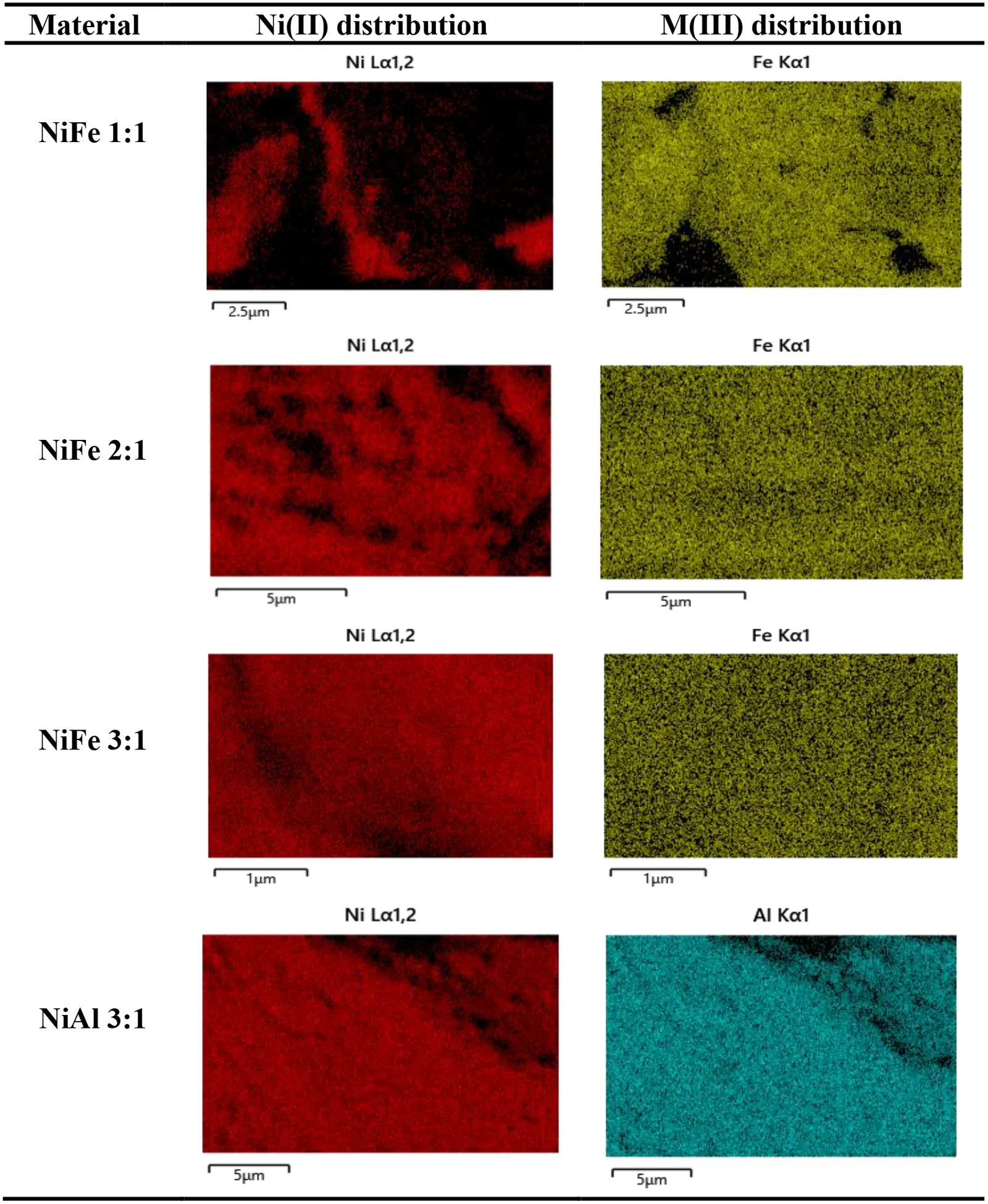
Element mapping for catalysts.

**Table 4:** Elemental Analysis for EDS

	% wt				
Material	Ni	Fe	Al	O	C
NiFe 1:1	11.99	28.41	-------	56.18	-------
NiFe 2:1	32.67	19.34	-------	47.99	-------
NiFe 3:1	52.66	16.93	-------	30.41	-------
NiAl 3:1	39.17	--------	8.26	47.34	5.23

#### 3.1.4 BET Analysis

The N_2_ adsorption–desorption isotherms of all materials exhibited a type III isotherm with an H3-type hysteresis loop, characteristic of lamellar structures and slit-shaped mesopores (Figure 7). NiFe 1:1 showed the highest BET surface area (121.5 m^2^/g), followed by NiAl 3:1 (84.5 m^2^/g), while NiFe 2:1 exhibited the lowest value (35.0 m^2^/g); these differences for the NiFe 2:1 material and the crystallite size (19.85 nm) could be correlated with nanosheet segregation leading as well to a lower pore volume. Materials with higher surface areas and smaller pore diameters (8–10 nm) exhibited improved removal performance, suggesting that surface area and pore structure play important roles in adsorption and catalytic activity (see Table 5).

**Figure 7:**
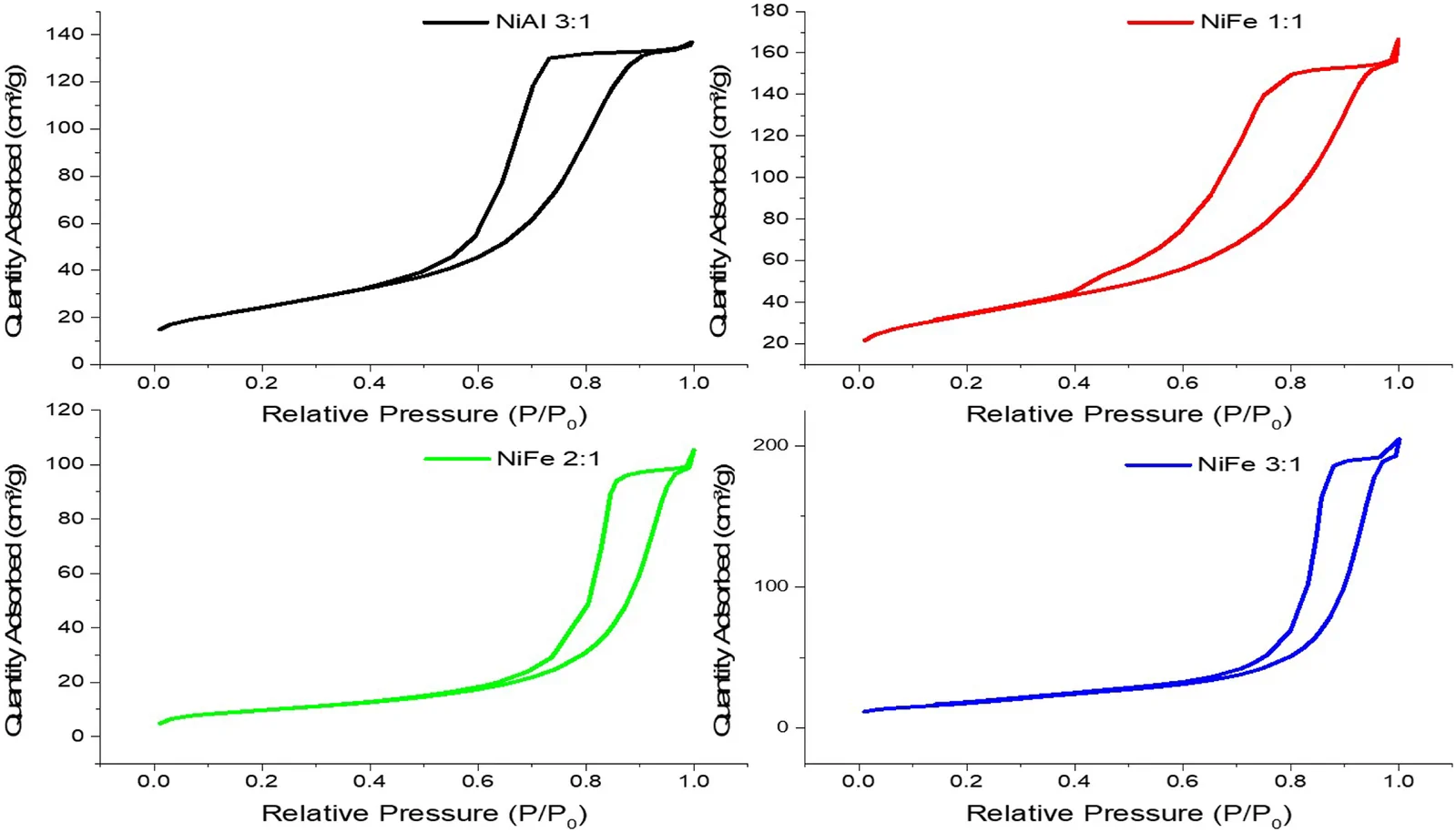
Isotherm of the studied materials.

**Table 5:** Textural and Surface Properties

Material	BET Surface Area (m^2^/g)	Pore Volume (cm^3^/g)	Pore Size (nm)
NiFe 1:1	121.50	0.24	8.0
NiFe 2:1	35.00	0.15	17.5
NiFe 3:1	63.38	0.30	18.8
NiAl 3:1	84.50	0.21	9.6

The apparent discrepancy between the crystallite size and the BET surface area of NiFe 2:1 can be attributed to differences in nanosheet stacking and pore accessibility rather than to crystallite size alone. The Scherrer crystallite size represents the coherent diffraction domain and does not directly reflect the particle size or accessible surface area. For lamellar materials such as LDHs, smaller crystallites may undergo denser stacking or stronger aggregation, reducing interlamellar pore accessibility and resulting in a lower BET surface area, despite their smaller diffraction-domain size. The compact morphology observed for NiFe 2:1 in the SEM images is consistent with this interpretation and may also explain its lower pore volume compared to that of NiFe 1:1.

### 3.2 Ciprofloxacin Removal

Figure 8 presents the ciprofloxacin removal profiles obtained with synthesized catalysts. In all cases, a rapid decrease in the ciprofloxacin concentration was observed during the first 20 min, followed by a progressively slower removal rate. After 120 min, NiFe-LDH(1:1) exhibited the highest overall removal, reaching approximately 87%. This result highlights the influence of the Ni/Fe composition on the overall removal performance.

**Figure 8:**
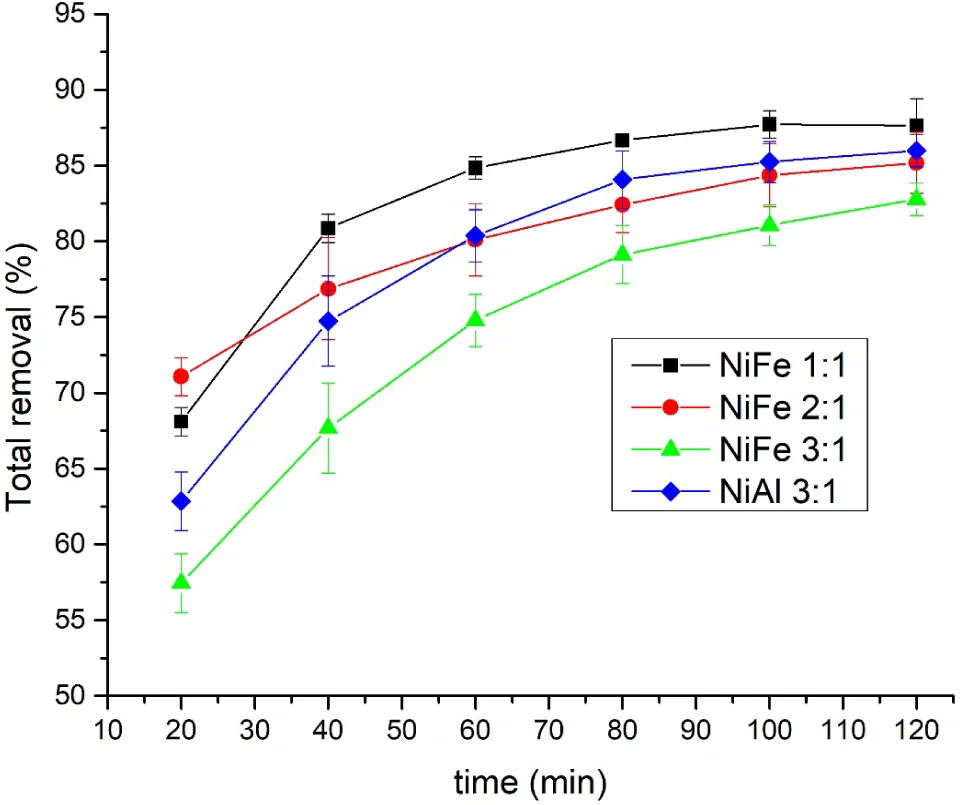
Time-course total removal profiles of ciprofloxacin via persulfate activation using NiFe and NiAl LDHs at different molar ratios.

Interestingly, NiFe-LDH(1:1) exhibited the highest overall removal despite containing the lowest Ni content among the NiFe series. This observation indicates that the overall performance cannot be explained solely by the amount of Ni present but rather by the specific Ni/Fe ratio and the resulting local chemical and electronic environment. Increasing the Ni ratio progressively decreases the relative contribution of Fe within the layered structure, which may influence the interaction with persulfate and subsequent oxidative transformation of ciprofloxacin.

The observed ciprofloxacin removal can involve two simultaneous processes: adsorption onto the catalyst surface and oxidative transformation promoted by persulfate activation. Therefore, the overall removal should not be interpreted directly as catalytic degradation. On the basis of the adsorption controls, the NiFe system exhibited approximately 30% removal attributable to adsorption at equilibrium, while the remaining removal was associated with the persulfate-dependent process, corresponding to approximately 57% oxidative removal. In contrast, NiAl-LDH exhibited a higher adsorption contribution of approximately 48%, whereas persulfate-dependent removal accounted for approximately 37%. These results indicate that adsorption contributes substantially to the apparent removal, particularly for the NiAl system, and that the two materials may differ in the relative contribution of adsorption and oxidative transformation.

Given these differences, NiAl-LDH was subsequently used as a reference system to evaluate whether the presence of redox-active Fe^3+^ provides an additional contribution to persulfate activation compared with that of a structurally related LDH containing the redox-inactive trivalent cation Al^3+^.

#### 3.2.1 Synergistic Effect of Fe­(III) Species

Ni/Fe and Ni/Al catalysts (both with a 3:1 molar ratio) were compared to determine whether the coexistence of two redox-active metals produces a synergistic enhancement in persulfate activation. The performance of the Ni/Fe catalyst was compared with that of Ni/Al, which contains Al as a non-redox-active metal (Figure 9).

**Figure 9:**
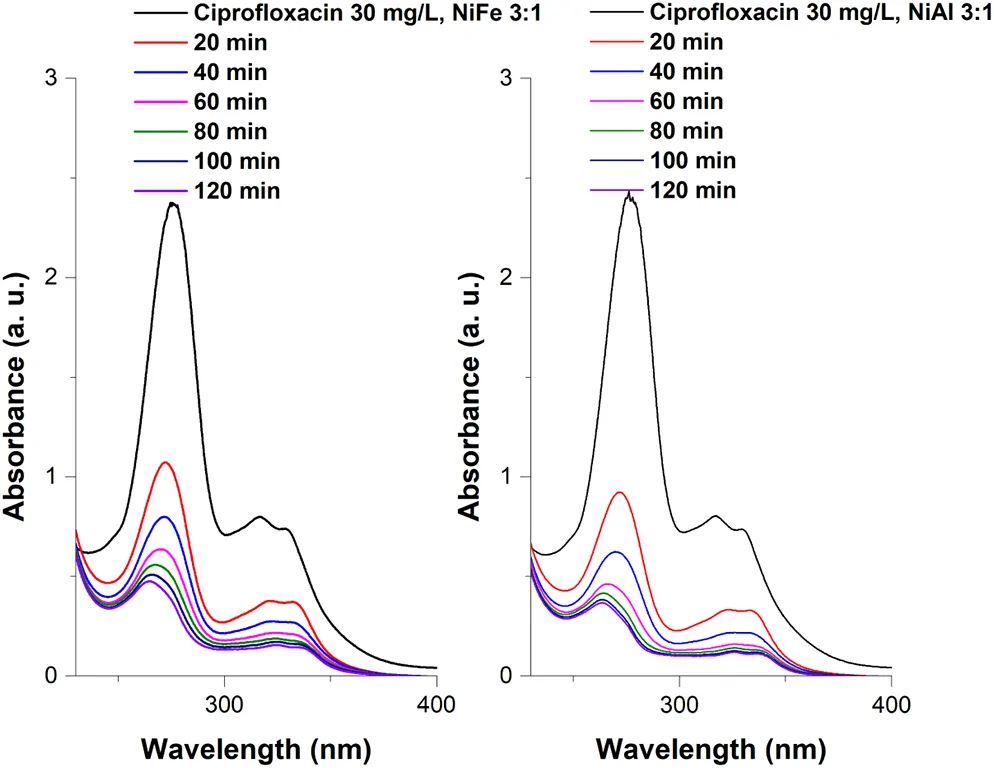
Removal profiles of the NiFe and NiAl systems.

UV–vis spectral monitoring confirmed a continuous decrease in the characteristic ciprofloxacin absorption band at 270 nm, with no emergence of new signals, suggesting the absence of stable transformation products within the monitored spectral range. This behavior is consistent with extensive structural disruption of the quinolone chromophore through an advanced oxidative degradation pathway.

The results indicate that NiAl-LDH exhibited comparable or slightly superior removal efficiency relative to NiFe-LDH at equivalent molar ratios (3:1) despite the absence of a redox-active trivalent metal. This behavior suggests that Fe^3+^ does not provide a significant synergistic contribution to persulfate activation under the conditions studied. Instead, the catalytic performance appears to be primarily governed by the Ni^2+^ centers and the structural properties of the LDH framework rather than by redox cycling between Fe^2+^/Fe^3+^. These findings are further supported by radical scavenger experiments, which revealed distinct mechanistic pathways between the two systems, and by DFT calculations, which showed that the electronic density relevant to persulfate activation is predominantly localized on Ni sites in both materials.

Although the removal efficiency reported here is comparable to that of more complex systems, the present work offers several practical advantages. The catalysts were synthesized via a simple coprecipitation route without postfunctionalization, and the treatment was conducted under mild conditions, at room temperature, without UV irradiation or external energy input, using a higher initial ciprofloxacin concentration (30 mg/L) than most reported systems. These features highlight the potential of Ni-based LDHs as cost-effective and scalable catalysts for persulfate-based water treatment (Table 6).

**Table 6:** Comparison of Ciprofloxacin Removal Efficiency among Various Advanced Oxidation and Bimetallic Catalytic Systems

Material	Conditions	Removal (%)	Reference
Alginate/TiO_2_ hydrogel	Photocatalysis (UV lamp (Evoluchem LED, 365 nm, 18 W)	100	^25^
	C_0_ = 20 mg/L		
	Reaction time: 300 min		
	Catalyst dosage:2 g		
	Room temperature		
	Magnetic stirring		
ZnO/GO nanocomposites	Photocatalysis (solar radiation)	99	^26^
	C_0_ = 10 mg/L		
	Reaction time: 75 min		
	Catalyst dosage: 20 mg		
	Room temperature		
	Magnetic stirring		
g-C_3_N_4_/BiOCl heterojunction	Persulfate activation and 300 W xenon lamp	86.5	^27^
	C_0_ = 10 mg/L		
	Reaction time: 90 min		
	Catalyst dosage: 30 mg		
	Room temperature		
	Magnetic stirring		
Co-doped Bi_2_Fe_4_O_9_	Peroxydisulfate activation and 300 W xenon lamp	84	^28^
	C_0_ = 10 mg/L		
	Reaction time: 70 min		
	Catalyst dosage: 20 mg		
	Room temperature		
	Magnetic stirring		
Double layered hydroxides NiFe and NiAl	Persulfate activation	87	This work
	C_0_ = 30 mg/L		
	Reaction time: 120 min		
	Catalyst dosage: 20 mg		
	Room temperature		
	Magnetic stirring		

#### 3.2.2 MS Analysis

High-resolution mass spectrum analysis was performed using a Thermo Scientific Orbitrap Exploris 120 in direct infusion mode (scan range: *m*/*z* 40–500; sheath gas: 45; auxiliary gas: 25; ion transfer tube temperature: 320 °C; vaporizer temperature: 275 °C).

Mass spectrometry was performed on a ciprofloxacin solution (30 mg/L) prepared from a commercial tablet. Aliquots collected at 20 and 120 min after treatment were centrifuged and filtered through a 0.22 μm disk. The initial solution exhibited the protonated molecular ion of ciprofloxacin (*m*/*z* 332), along with additional signals at *m*/*z* 274, 166, and 135, which may correspond to tablet excipients or formulation-related compounds, as they were also present in untreated samples. After 20 min of treatment, the ciprofloxacin parent ion was almost completely suppressed, while the *m*/*z* 274 peak remained constant; at 120 min, the *m*/*z* 332 signal was only faintly detectable. In contrast, studies on electro-Fenton degradation of ciprofloxacin have reported several intermediate ions close to the parent mass (e.g., *m*/*z* 154, 164, 190, 263, 344, 362).^29^ In the present work, none of these signals were observed, highlighting the distinctive pathway of the NiFe/PS system, which appears to favor advanced degradation rather than the persistence of partially degraded species (Figure 10).

**Figure 10:**
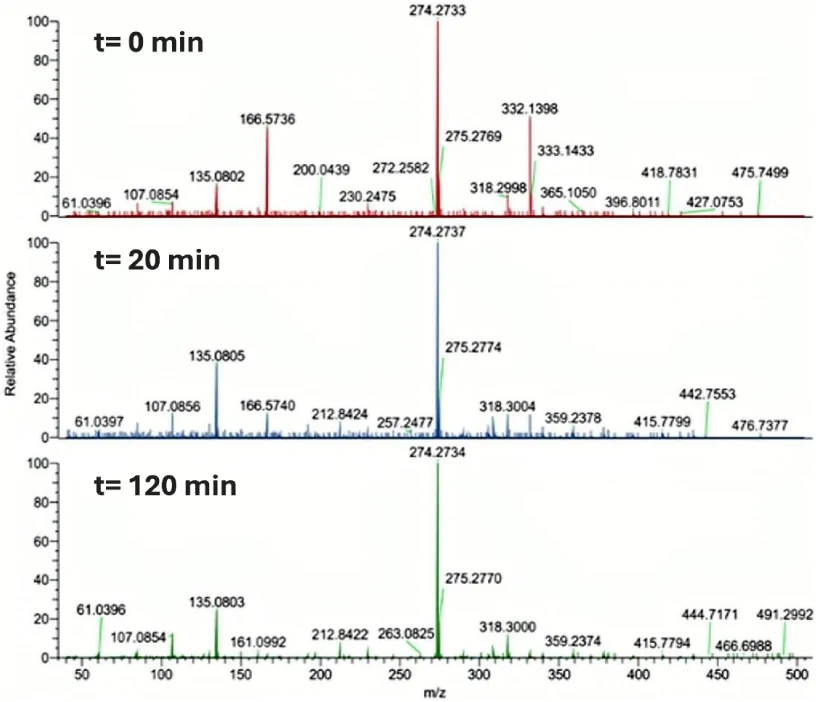
High-resolution mass spectra monitoring the temporal evolution of ciprofloxacin and the suppression of intermediate metabolites at 0, 20, and 120 min.

#### 3.2.3 Experimental Design

The 2^2^ factorial design, performed in triplicate, revealed that ciprofloxacin removal efficiency was significantly influenced by catalyst dosage (p-value = 0.028 < 0.05), while persulfate volume did not show a statistically significant effect (p-value = 0.294 > 0.05). The model adequately described the experimental data (R^2^ = 0.998), where the factor levels are defined as described in Table 1.

The empirical model obtained is expressed as

Removal(%)=73.177+6.558×(catalyst dosage)−0.588×(persulfate volume)

The main effect plot (Figure 11a) confirms the dominant role of catalyst dosage, which is consistent with the proposed surface-mediated persulfate activation mechanism. In contrast, increasing persulfate volume produced a slight negative trend in removal efficiency (Figure 11b), possibly due to radical self-quenching or nonproductive oxidant consumption at higher oxidant concentrations.

**Figure 11:**
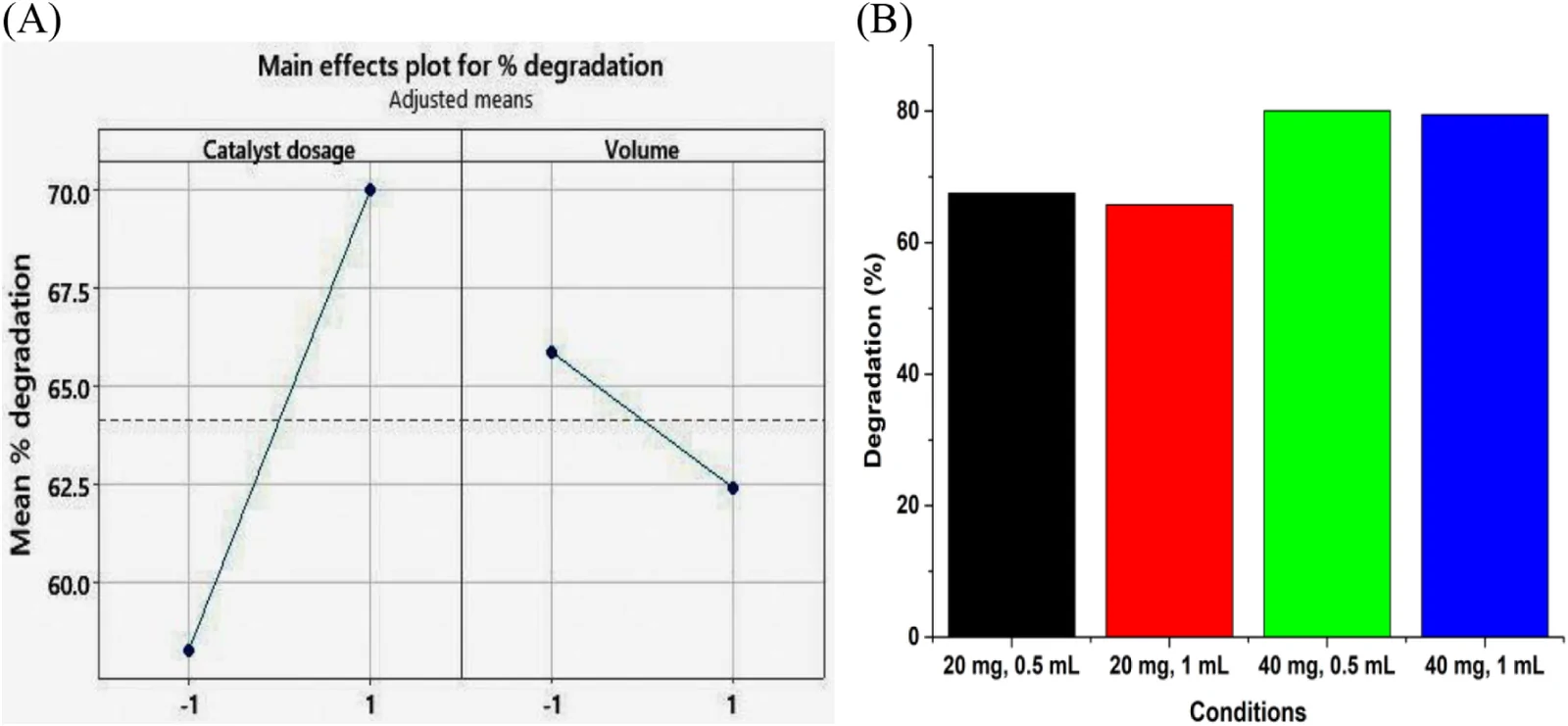
(a) Main effects plot for % removal and (b) effect of catalyst dosage and persulfate volume on ciprofloxacin removal efficiency.

#### 3.2.4 Radical Scavenger Experiments and Mechanistic Insights

Radical scavenger experiments were conducted at 20 min to compare the degradation behaviors of the NiFe 3:1 and NiAl 3:1 systems. The tests included adsorption-only conditions, persulfate activation, methanol (MeOH), and *tert*-butyl alcohol (TBA). The results indicate that the trivalent metal plays a key role in the degradation pathways.

For the NiFe system, ciprofloxacin removal increased from 26.6% under adsorption-only conditions to 49.3% in the presence of persulfate. However, the addition of TBA reduced removal to 43.2%, whereas MeOH increased it to 47.9%. The greater inhibition observed with TBA compared to MeOH suggests that hydroxyl radicals (OH•) are the predominant reactive species, since TBA selectively quenches OH• while MeOH scavenges both OH• and SO_4_•–, suggesting the participation of hydroxyl radicals and a radical-mediated Fenton-like mechanism.

In contrast, the NiAl system exhibited high adsorption (48.2%) and similar removal efficiencies under all conditions, with 55.5% in the presence of persulfate, 55.3% with MeOH, and 54.3% with TBA. The minimal variation observed in the presence of scavengers indicates that the degradation pathway in NiAl 3:1 is less dependent on free radical species in solution and more influenced by adsorption and surface-mediated processes (Figure 12).

**Figure 12:**
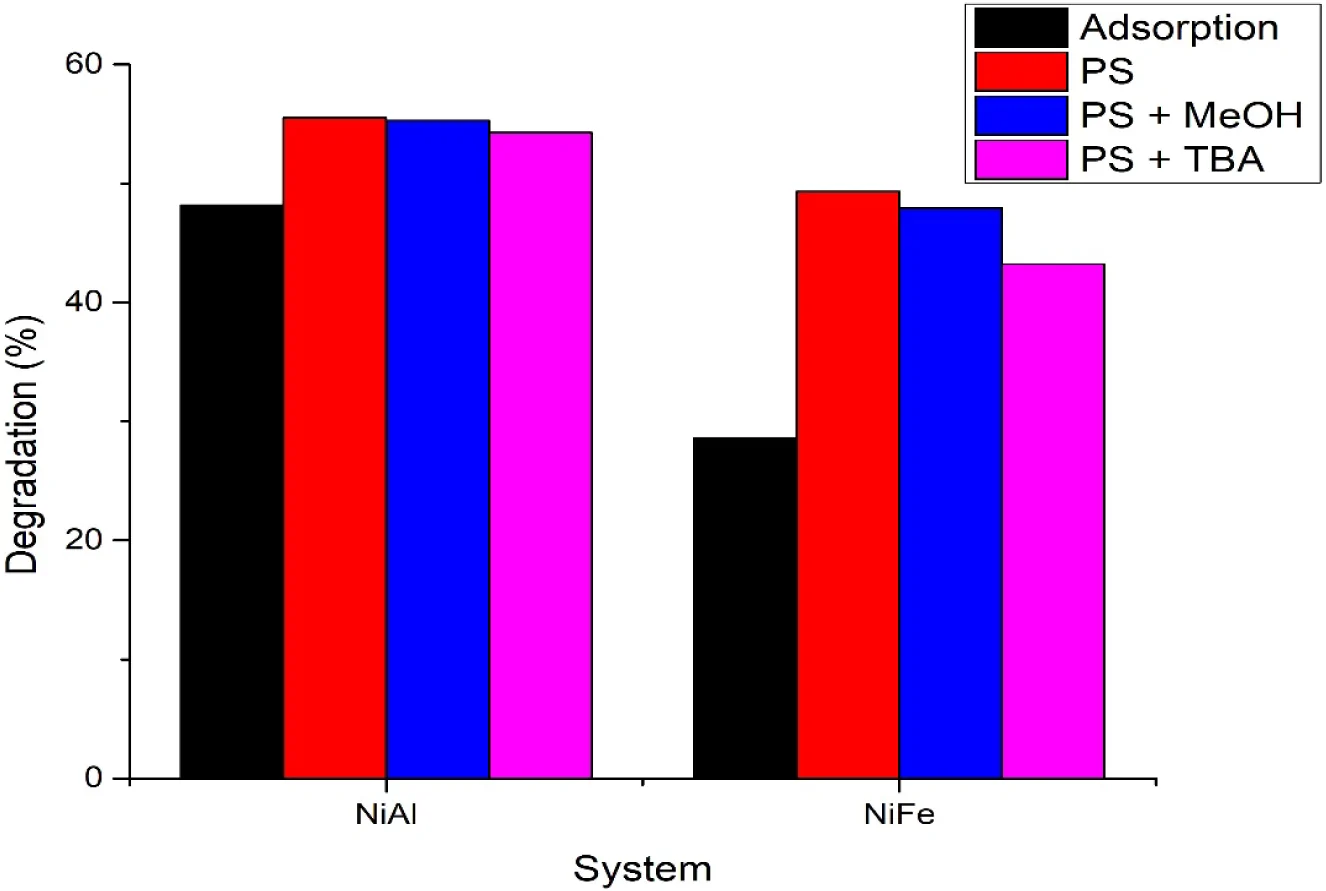
Effect of radical scavengers on ciprofloxacin removal at 20 min.

To put our findings into perspective, recent advanced oxidation studies have primarily focused on conventional redox-active centers to trigger persulfate activation. For instance, dual-atom catalysts such as Cu–Fe systems rely heavily on cyclic transition-metal redox couples to drive the homolytic cleavage of the oxidant.^30^ In sharp contrast, our NiFe- and NiAl-LDH frameworks demonstrate that persulfate activation can be highly optimized through structural electronic modulation induced by the neighboring trivalent cations (Fe^3+^ or Al^3+^) rather than relying solely on traditional redox synergy.

The overall relationship among crystallinity, BET surface area, crystallite size, morphology, and ciprofloxacin removal suggests that BET surface area plays an important role in the removal process using layered double hydroxides, as adsorption may promote the subsequent catalytic process. Among the materials evaluated, NiFe (1:1) exhibited the highest removal, followed by NiAl, which showed the second-highest BET surface area and overall removal. This interpretation is consistent with the pore volume and pore diameter results, as these two materials exhibited similar values for both parameters. This behavior is consistent with the general types of persulfate activation radical and no radical where the material surface plays a key role in reactive adsorption.

#### 3.2.5 Kinetics of Ciprofloxacin Removal

The removal kinetics were analyzed using pseudo-first-order (eq 6) and pseudo-second-order (eq 7) models. The apparent kinetic constants and corresponding R^2^ values are summarized in Table 7.

**Table 7:** Kinetic Parameters of the Materials Studied for Ciprofloxacin Removal

Material	K_1_ (min^–1^)	R^2^	K_2_ (A^–1^·min^–1^)	R^2^
NiFe 1:1	0.0023	0.7908	0.0256	0.9473
NiFe 2:1	0.0016	0.9469	0.0151	0.9974
NiFe 3:1	0.0029	0.9275	0.0157	0.9923
NiAl 3:1	0.0027	0.8778	0.0218	0.9817

For all materials, the pseudo-second-order model provided a superior fit to the experimental data compared to the pseudo-first-order model. Although comparable kinetic trends were observed among the catalysts, NiFe 1:1 exhibited the highest apparent rate constant (*k*_2_ = 0.0256 A^–1^·min^–1^), consistent with its superior overall removal efficiency. These results suggest that surface-mediated interactions play a significant role in the overall removal process. The dominance of the pseudo-second-order model, together with the scavenger experiments, indicates that adsorption and surface-mediated processes contribute substantially to ciprofloxacin removal, particularly in the NiAl system where radical participation was minimal.

3 $$\ln\left(\frac{A_{t}}{A_{0}}\right)=-k_{1}t$$

4 $$\frac{1}{A}=k_{2}t+\frac{1}{A_{0}}$$

#### 3.2.6 DFT Analysis of Persulfate Interaction with Ni-Based LDH Models

DFT calculations were performed to investigate the relationship between catalytic performance and the electronic environment of the Ni-based LDH systems by comparing cluster models representing NiAl-LDH and NiFe-LDH and their interaction with persulfate. The analysis included HOMO and LUMO distributions, HOMO–LUMO energy gaps, charge transfer, and the interaction energies of persulfate with the cluster models. These calculations provide complementary molecular-level information to the experimental observations and help assess the different interactions promoted by the redox-active Fe^3+^ and the redox-inactive Al^3+^ centers.^31^

Experimentally, NiAl-LDH exhibited comparable or slightly higher overall ciprofloxacin removal than some NiFe-LDH compositions. However, the adsorption contribution was substantially higher for NiAl-LDH, whereas the persulfate-dependent contribution was more pronounced for the NiFe system. This distinction, together with the scavenger experiments, suggests that the two materials may not rely on the same dominant interaction pathways. A related observation was previously reported in our study of MgFe-, CoFe-, and NiFe-LDH catalysts,^32^ where the incorporation of a nonredox metal did not necessarily result in inferior catalytic performance. These results indicate that the catalytic behavior cannot be attributed solely to the number of redox-active metal centers and may instead depend on the local electronic and coordination environment of the active sites.

The isolated cluster models exhibited different electronic structures, with HOMO–LUMO gaps of 4.08 eV for the NiAl model and 3.02 eV for the NiFe model (Table 8). The lower gap calculated for the NiFe model indicates a more closely spaced frontier-orbital structure than that of NiAl, which may facilitate electronic interactions with persulfate. The HOMO distributions in both models were mainly localized around Ni–O environments (Figure 13a and 13c), with only a minor contribution from the trivalent metal centers. Similarly, the LUMO density was primarily distributed around oxygen-containing sites coordinated to Ni, indicating that the Ni–O environment plays an important role in the frontier electronic structure of both cluster models.

**Table 8:** DFT Result Data

Parameter	NiFe	NiAl
E_HOMO_ (eV)	–5.37	–5.33
E_LUMO_ (eV)	–2.35	–1.24
E_G_ (eV)	3.02	4.08
χ	3.86	3.29
η	1.51	2.04
S	0.66	0.49
ΔG_ads_ (kcal/mol)	–100.38	–63.68
Q|e|	–2.45	–1.69
E_G_ after persulfate adsorbed (eV)	3.03	3.84

**Figure 13:**
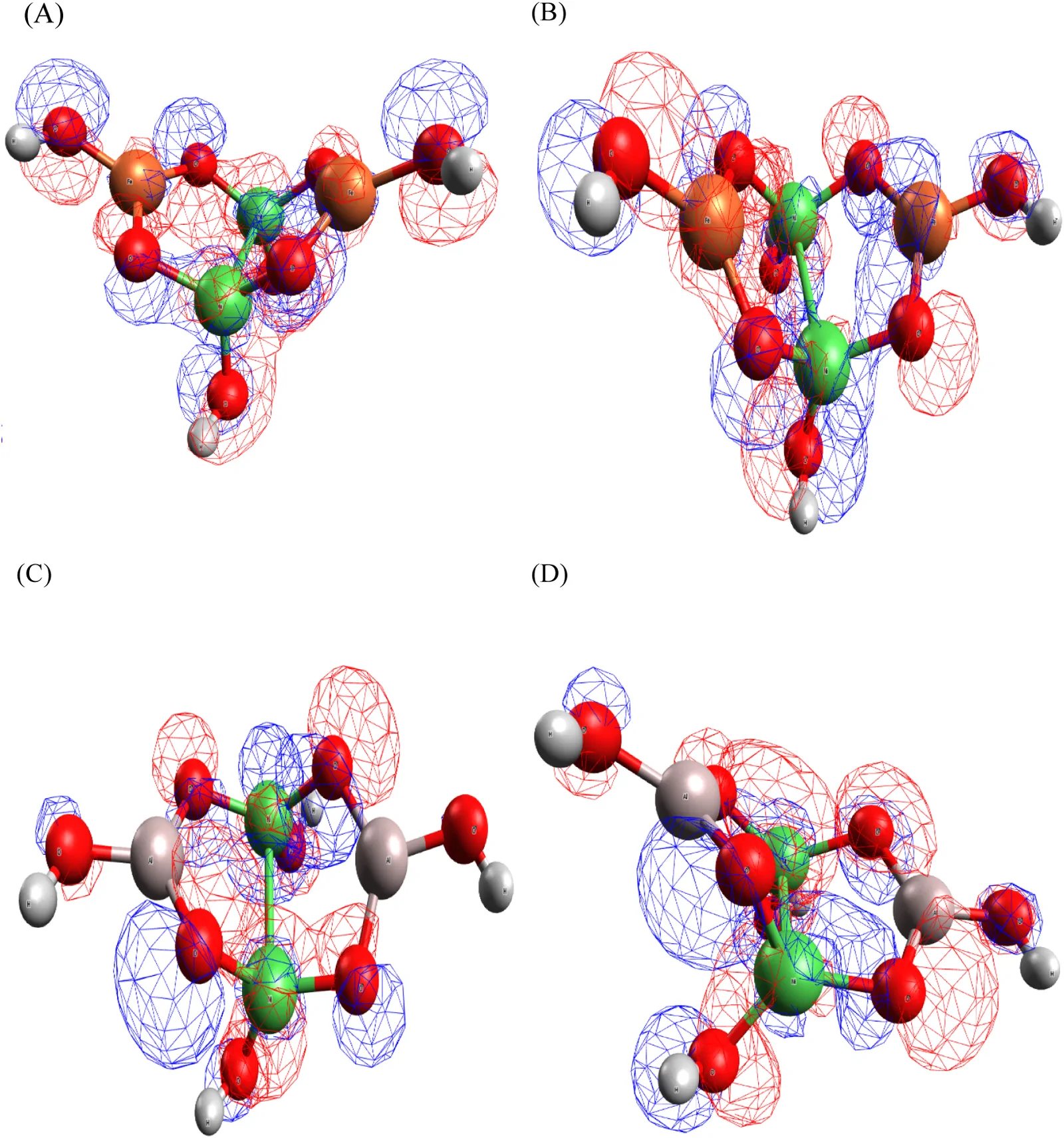
Representation of HOMO: (A) HOMO NiFe, (B) LUMO NiFe, (C) HOMO NiAl, and (D) LUMO NiAl.

A marked difference was observed after persulfate interaction. In both models, optimization led to complete cleavage of the O–O bond of persulfate, indicating a strong chemical interaction rather than a simple molecular adsorption process (Figure 14). The NiFe model exhibited a substantially more favorable interaction energy (−100.38 kcal mol^–1^) compared with the NiAl model (−63.68 kcal mol^–1^), together with a larger net charge transfer to the persulfate-derived species (−2.45 and −1.69 e, respectively). Despite this stronger interaction, the NiFe cluster largely preserved its initial structural framework, whereas the NiAl cluster underwent a more pronounced structural reorganization after persulfate interaction (Figure 14a and b). These results indicate that Fe^3+^ and Al^3+^ modify the interaction of the Ni–O framework with persulfate in different ways.

**Figure 14:**
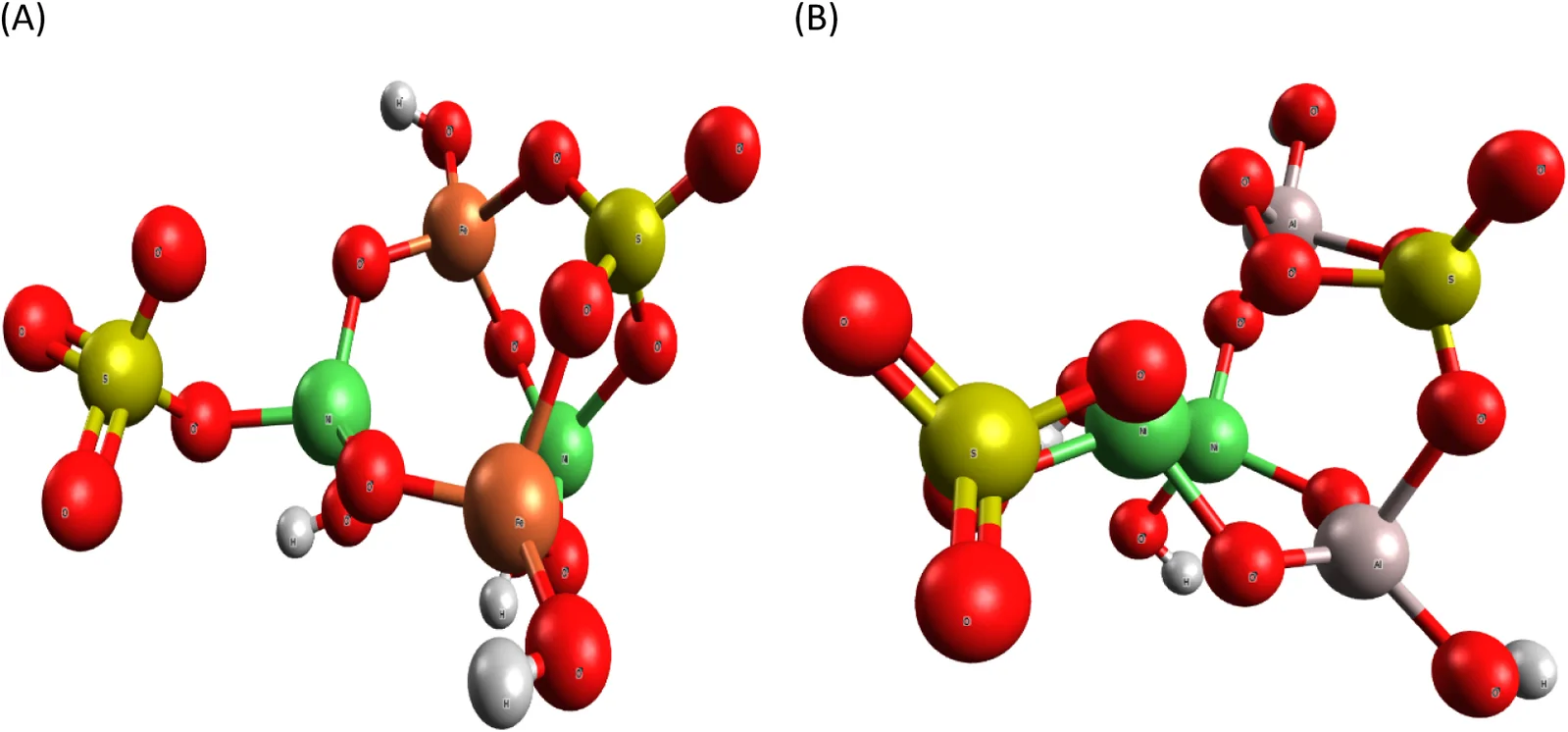
S_2_O_8_^–2^ adsorption on (A) NiFe- and (B) NiAl-LDH models.

The calculated results are consistent with the experimental evidence obtained from the scavenger experiments, which indicated a larger relative contribution of adsorption in the NiAl system and a more pronounced catalytic contribution in the NiFe system. The stronger persulfate interaction and greater charge redistribution calculated for the NiFe model provide molecular-level support for a more effective activation of persulfate in the Ni–O/Fe-containing environment. In contrast, the greater structural reorganization observed for the NiAl model suggests a different interaction pathway. Although these calculations do not by themselves establish whether the subsequent oxidation proceeds exclusively through radical or nonradical species, they demonstrate that the nature of the trivalent metal significantly modifies the electronic and structural response of the Ni-based LDH to persulfate.

## 4 Conclusion

Ni-based layered double hydroxides were successfully synthesized and evaluated as catalysts for ciprofloxacin degradation via persulfate activation. Among the studied materials, NiFe(1:1) exhibited the highest removal efficiency, highlighting the importance of metal composition in catalytic performance. However, increasing the Ni content did not improve activity, indicating that catalytic behavior is not directly governed by the number of active sites.

A key finding of this work is that no significant redox synergy between Ni and Fe was observed. The comparable or superior performance of NiAl-LDH demonstrates that the presence of a second redox-active metal does not necessarily enhance persulfate activation. These results challenge the conventional assumption that bimetallic redox interactions are the primary factors driving catalytic efficiency in LDH systems.

Additionally, the results support that the removal process presents two steps, with adsorption being fundamental to reaching the catalytic step, as observed in the scavenger tests. Fe-based LDH materials exhibit more catalytic behavior, while the Al-based material leads to a more adsorptive removal pathway. In this context, morphological and textural characteristics are relevant factors in determining the overall performances of these systems.

DFT calculations, supported by radical scavenger experiments, revealed that Ni sites dominate persulfate activation while the trivalent metal plays a secondary role by modulating the electronic structure and reaction pathway. This highlights the importance of electronic effects over simple redox considerations in determining the catalytic performance.

Overall, this work provides new insight into the design of LDH-based catalysts, emphasizing that electronic-structure tuning is a more effective strategy than increasing the number of redox-active components, enabling efficient pollutant removal under mild conditions without external energy input. These findings provide a basis for the rational design of more efficient LDH-based catalysts for environmental remediation.

## Data Availability

The data sets generated and analyzed during the current study are available from the corresponding author upon reasonable request.
